# Femtosecond Laser-assisted Allogenic Additive Stromal Keratoplasty With or Without Excimer Laser Donor Keratomileusis for Management of Keratoconus

**DOI:** 10.18502/jovr.v16i4.9761

**Published:** 2021-10-25

**Authors:** Mohammad-Reza Jafarinasab, Yasaman Hadi, Goldis Espandar

**Affiliations:** ^1^Ophthalmic Research Center, Research Institute for Ophthalmology and Vision Science, Shahid Beheshti University of Medical Sciences, Tehran, Iran; ^2^Eye Research Center, Rassoul Akram Hospital, Iran University of Medical Sciences, Tehran, Iran

**Keywords:** Allogenic, Ectasia, Keratoconus

## Abstract

We describe a modified allogenic intrastromal lenticule implantation technique for management of keratoconus (KCN). Patients with advanced KCN already scheduled for corneal transplantation were enrolled. An allogenic corneal lenticule was implanted inside a stromal pocket created by femtosecond laser. In three cases, the estimated refractive error of the recipient eyes was corrected on the donor lenticules using an Excimer laser. All operated eyes underwent corneal crosslinking at the time of surgery. This method was named “Femtosecond Laser-assisted Allogenic Stromal Keratoplasty Without and With Excimer Laser-assisted Donor Keratomileusis”; briefly called FASK and FASK Plus EDK, respectively. Two out of five patients were satisfied with the results. There was a decrease in the average simulated keratometric values as well as myopia when FASK Plus EDK was performed. Increased corneal thickness was achieved in all cases. Graft edema gradually decreased over weeks but interface wrinkling and lenticule folds in the visual axis remained as a problem during follow-up period. No other complications were encountered.

##  INTRODUCTION 

Keratoconus (KCN) is the most common primary corneal ectasia characterized by non-inflammatory, slowly progressive thinning and steepening of the central or paracentral part of the cornea of unknown etiology, resulting in irregular astigmatism and progressive decrease in quality of vision.^[1,2]^

Depending on the diagnostic criteria and different diagnostic tools employed, estimation of the incidence and prevalence of KCN in any given population is different.^[3,4,5]^

Management of KCN in the early stages includes use of spectacles, rigid gas permeable contact lenses, and intracorneal ring segments (ICRS) implantation.^[6,7,8,9]^ In cases with severe irregular astigmatism where spectacles or contact lenses are not tolerated, lamellar or penetrating keratoplasty is usually considered.^[6]^ Corneal collagen cross-linking (CXL) has been shown to be effective in slowing down or arresting the progression of KCN.^[10]^

Since corneal transplantation is costly and an invasive procedure entailing possible complications, treatments that may replace or postpone corneal transplantation are a desirable solution and an ongoing subject of research.

In recent years, synthetic intrastromal corneal ring segments (ICRS) have proven effective treatment for ectatic corneal disorders. ^[9]^ Despite favorable topographic and visual outcomes, there are risks associated with implanting a synthetic substance within the cornea. Complication rates up to 30% have been reported in some series ^[9]^ including misalignment, migration, extrusion, corneal perforation, infection, and tissue reactions. ^[9,11,12,13]^

From the point of biocompatibility, use of biological inlays, derived from allogenic donor corneas may offer advantages over synthetic ICRSs and inlays.^[12,13]^ In recent years application of stromal tissue addition for potential management of corneal ectasia with or without CXL has been a hot topic in some studies.^[14,15]^ The aim of the current pilot study was to introduce a modified allogeneic inlay for management of KCN and report the feasibility, refractive outcomes, and possible complications.

### Patient Selection

From October 2015 to January 2016, five consecutive patients with advanced KCN at the Cornea and Refractive Surgery Service of Labbafinejad Medical Center already scheduled for lamellar or penetrating keratoplasty were enrolled. Patients with history of corneal surgery or any corneal pathology other than KCN and patients with central corneal thickness < 400 microns or opacities were excluded. The study was approved by the Ethics Committee of the Ocular Tissue Engineering Research Center at Shahid Beheshti University of Medical Sciences. After explaining the procedure and possible complications, informed consent was obtained from all patients.

##  SURGICAL METHOD 

### Pocket Creation

Under topical anesthesia, in the usual sterile manner, a stromal pocket was created using the lamellar keratoplasty mode of the TechnolasⓇ Femtosecond Workstation (Technolas Perfect Vision, Bausch and Lomb, Munich, Germany). The energy setting and spot distance were 1600 nJ and 4.1/4.1 µm, respectively. The depth of the pocket was set at 75% depth of the thinnest point (300–400 μ) and the pocket diameter was selected 2.50 mm smaller than the vertical corneal diameter. Two pocket entries 4 and 5 mm in size were created on the steepest corneal topographic axis 180° away using the astigmatism keratotomy mode.

### Donor Preparation

Prepared corneal allograft tissues from the Iranian Eye Bank, (Eye Bank of the I.R. Iran) eligible for DALK (Type I) or DSAEK (Type II) were employed. The epithelium and Descemet's membrane (DM) of donors prepared for DALK (Type I) were removed. In cases where precut tissue suitable for DSAEK was used (Type II), the posterior lamella had already been used for DSAEK and we made sure that the anterior free cap was completely de-epithelialized. Both type I and type II corneal tissues were punched using the Hessberg's punch at a diameter 20% smaller than that of the pocket.

### Correction of Refractive Errors

For patients #2 and 5, no refractive correction was performed on the lenticule while in the other three subjects we decided to correct the refractive error as much as possible on the donor lenticule before insertion. First, we registered the patient's eye for the planned correction and manually marked reference points at 3, 6, and 9 clock hour positions. The donor lenticule was placed over the cornea and marked exactly at the same locations as the patient's cornea. The lenticule was then removed and a soft contact lens (14 mm diameter) was fitted to protect the recipient cornea from unwanted ablation. The lenticule was put back on the contact lens and its position was adjusted aligning the preset marks. Laser ablation was subsequently performed on the lenticule using the Allegretto EX500 excimer laser machine (WaveLight Allegretto Wave Eye-Q laser devices; ALCON Laboratories, USA).

### Preparation for CXL 

Both the donor lenticule and the recipient cornea underwent epithelium on, CXL, after soaking the lenticule in riboflavin 0.1% riboflavin in 20% dextran T500 and injection of the same material into the recipient corneal pocket for 30 min.

### Insertion of the Lenticule into the Pocket

The femtosecond laser-created arcuate incisions and intrastromal pockets were gently dissected using a blunt dissector. The riboflavin soaked lenticule was inserted into the pocket and its meridian of orientation was matched with meridian of the recipient cornea using the preset marks. The incisions were secured using 10-0 nylon sutures.

### Completion of CXL

CXL was completed using UV fluence of 9mW/cm ${}^{2}$ for 10 min to obtain a total energy of 5.4 J/cm ${}^{2}$ .

### Post operation Medications and follow-up

Postoperatively, patients were prescribed betamethasone 0.1% eye drops (Sina Darou, Tehran, Iran) four times a day, non-preserved levofloxacin 0.5% eye drops (Sina Darou, Tehran, Iran) four times a day, and non-preserved artificial tears Artelac (Baush and Lomb, Rochester, NY USA) four to six times a day. Levofloxacin drops were discontinued in seven days and betamethasone drops tapered off over six weeks. Sutures were removed no later than four weeks after surgery. Patients were visited regularly on the first day, first week, and first month after surgery and then every three months. At each visit, they underwent complete ophthalmic examinations, including refraction, corrected distance visual acuity (CDVA) and uncorrected distance visual acuity (UDVA) measurements, intraocular pressure monitoring, and thorough slit lamp examination.

##  RESULTS

**Figure 1:**
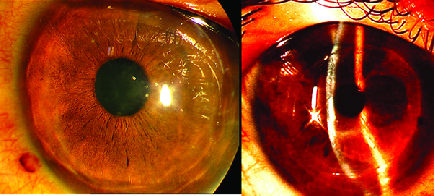
Slit photo of case number 5, 51 months after FASK shows mild haziness and wrinkling of interface.

Five eyes of five patients with advanced KCN were operated. Mean age of patients was 29 (range, 20 to 46) years. Mean follow-up was 28.4 (range 8 to 55) months. Patients' demographics and follow-up periods are presented in Table 1. Table 2 contains pre- and postoperative visual acuity, refractive and keratometric data for each individual. Patients number 2, 3, and 4 were not happy with the final visual outcome and underwent DALK after 8, 12, and 18 months, respectively. Patient number 1 and 5 were satisfied with the results and were willing to wait and try topography-guided photorefractive keratectomy. In terms of safety, only one patient (number 3) lost more than one line of CDVA. No intraoperative or postoperative complications such as stromal allogenic rejection, infection, or progressive corneal haze were observed except for early graft edema which decreased gradually in addition to wrinkling and folds in the lenticule and recipient cornea [Figure 1]. There was a decrease in the average simulated keratometric values in cases 1, 3, and 4 in contrast to cases 2 and 5. Corneal thickness was increased in all patients.

**Table 1:** Demographics and follow up time


**Case number**	**Age**	**Sex**	**Eye**	**F/u(m)**	**Final plan**
**1**	21	F	OD	55	T-CAT
**2**	46	F	OD	8	DALK
**3**	29	M	OS	12	DALK
**4**	20	M	OS	16	DALK
**5**	31	M	OS	51	T-CAT
T-CAT, topography-guided custom laser ablation; DALK, deep anterior lamellar keratoplasty					

**Table 2:** Pre- and postoperative visual, refractive, and keratometric values


	**UCDVA**	**CDVA**	**Refraction(D)**	**SimKs(D)**	**SimKf(D)**	**SimK/AVG(D)**	**Type of surgery**
1	Pre	20/800	NI	Irregular	55.49@105	46.60@15	51.05	FASK-plus
	Post	20/120	20/50	–4.25–1.00@65	50.74@104	47.98@14	49.36	
	Diff	+5 lines	+7 lines	_	–4.75 D	+1.38 D	–1.64 D	
2	Pre	20/400	20/200	–9.25–4.4@50	49.99@144	46.94@54	48.7	FASK
	Post	20/400	NI	Irregular	56.50@73	54.5@163	55.5	
	Diff	0	–1 line	+6.51 D	+7.56 D	+6.8 D	
3	Pre	20/400	20/80	–6.25–1.75@175	48.75@75	46.5@165	47.12	FASK-plus
	Post	20/200	20/160	–0.25–1.75@105	49.5@165	45.00@75	45.25	
	Diff	+1 line	–2 line	+6.00 D	+0.75 D	–1.5 D	–1.87 D	
4	Pre	20/400	NI	Irregular	48.00@95	45.75@5	46.88	FASK-plus
	Post	20/300	20/200	–2.5–2.5@175	47.25@60	45.00@150	45.25	
	Diff	+1 line	_	–0.8 D	–0.75 D	–1.63 D	
5	Pre	20/80	NI	Irregular	54.90@45	50.50@135	52.7	FASK
	Post	20/200	20/40	–7.25–3.25@153	55.50@77	51.50@167	53.5	
	Diff	–3 lines	+3 lines	+0.6 D	+1.00 D	+0.8 D	
UCDVA, uncorrected distance visual acuity; CDVA, corrected distance visual acuity; Diff, difference; NI, no improvement; SimK, simulated keratometry; D, diopter; AVG, average; FASK, Femtosecond Laser-assisted Allogenic Stromal Keratoplasty			

**Figure 2:**
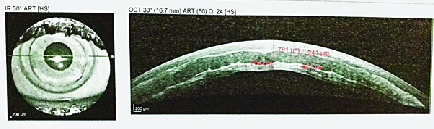
Anterior segment OCT of case number 2. Wrinkling of anterior and posterior interface can be seen.

**Figure 3:**
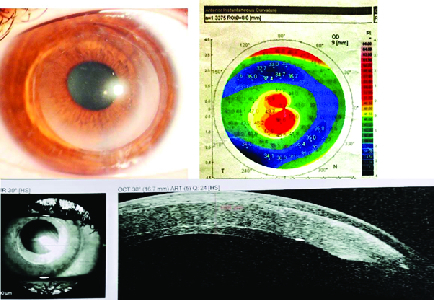
Preoperative topography, postoperative slit photo, and anterior OCT of patient number one. Faint haziness of visual axis without any interface wrinkling is seen 55 months after surgery.

The main cause for persistent visual dissatisfaction leading to corneal transplantation were wrinkles or folds in the lenticule and recipient cornea [Figure 2].

### Representative Case 

A 20-year old lady with advanced KCN in her right eye had severe distortion of the retinoscopy reflex and high levels of irregular astigmatism UCVA was 20/1000 which was not correctable. She had abandoned hard contact lenses because of dissatisfaction since last year.

UCVA of the left eye was 20/200 which was improved to 20/80 with spectacle correction of –8.0 –7.25@160 and to 20/30 with RGP contact lenses.

All the surgical options for the right eye including ICRSs and DALK were thoroughly discussed with the patient and she decided to try our new treatment option.

Preoperative simulated keratometry (SimK) in the right eye was 55.49@105/46.65@15 and topographic astigmatism was 8.84105.

A stromal pocket 9.0 mm in diameter and at 360 µ depth was created as described in the methods section. A full thickness corneal lenticule with a diameter of 7.5 mm was punched from a donor cornea (type I). After preparing the lenticule, based on topographic SimK, we corrected the refractive error including corneal astigmatism and myopia on the donor lenticule. We planned to achieve an average SimK of about 44.0 D postoperatively and correct six diopters of astigmatism. Calculated refractive error correction for this target was –5.50–6.00 @15 with SE of about –8.50 D. After lenticule insertion into the stromal pocket and CXL as described above, a silicone-hydrogel bandage contact lens (Bausch & Lomb Incorporated, Rochester, NY, USA) was placed on the cornea. On the first day after the operation, the lenticule was edematous and UCVA was about 20/400. Lenticule edema decreased over the next few weeks and UCVA was improved to 20/200 after one month. Refractive error 6 and 18 months after surgery was –2.50 –1.0@62 and –3.37 –2.0@30, respectively. CDVA improved to 20/60 18 months after surgery. Refraction and CDVA, 55 months after surgery were stable. Pre- and postoperative CCT were 487 and 727 microns, respectively. Preoperative topography together with postoperative slit photo and anterior OCT of the patient are represented in Figure 3.

##  DISCUSSION

The concept of tissue addition for correction of refractive errors was first introduced by Jose Barraquer in 1949.^[16]^ With the development of laser vision correction and synthetic intrastromal inlays, the Barraquer technique of epikeratophakia and keratomileusis were substituted by newer techniques, however, concerns were raised regarding diffusion of nutrients across the synthetic inlay.^[13]^ Recently, lenticules extracted from eyes undergoing SMILE have been used as autograft or allografts for correction of hyperopia based on Barraquer`s law of thickness.^[17,18,19,20]^

The lenticule harvested from a myopic-SMILE procedure is convex-shaped and can be used to steepen the central cornea of a hyperopic eye. By implanting a concave lenticule, inside a stromal pocket, the anterior corneal curvature can theoretically be reshaped to be less steep or less hyper-prolate, hence improving visual function. Even though autologous lenticule implantation can be considered in exceptional cases of significant antimetropia, in the vast majority of cases such lenticules should be synthetic or allogenic in nature.

Improving corneal irregularity in addition to correction of refractive errors and stromal thinning in advanced KCN using tissue addition remains a challenge. The method we have devised increases corneal thickness with the addition of allogenic stroma and also allows the refractive error to be addressed by reshaping the donor lenticule applying customized excimer laser ablation before implantation along with CXL, alternatively refractive error correction may be postponed after stabilization and be later performed on a thickened and cross-linked cornea.

We performed excimer laser ablation on the donor lenticule in an attempt to correct the refractive error in cases number 1, 3, and 4. In these three cases, postoperative average keratometry and consequently myopia were decreased whereas average keratometry increased in cases number 2 and 5 for whom laser refractive correction was not performed. Clinically, we noted no significant difference in terms of patient satisfaction regarding corrected visual acuity between subjects who received refractive corrected lenticules and those who received a non-modified lenticule [Table 2].

Biological inlays, proposed in the current pilot study, theoretically offer superior permeability and biocompatibility as compared to synthetic inlays. Lenticule implantation is essentially a selective lamellar keratoplasty procedure; hence it still carries the risk of rejection if the lenticule is not autogenic. However, the risk of rejection is expected to be low, as compared to full-thickness or lamellar corneal transplantation, because the antigenic load of purely stromal lenticule should be less than PKP or DALK due to absence of the more antigenic epithelium and endothelium.^[18]^ In addition, the intrastromal implanted lenticule may be better protected from immune reactions inside the recipient corneal pocket due to lack of exposure to tears and aqueous humor, and farther distance from the limbus.

Predictability of refractive results following lenticule implantation for KCN warrants investigation; however, our results show that correction of recipient refractive error on the donor lenticule could significantly reduce central corneal steepness which contrasts with lenticules with no refractive ablation. Thickness of implanted lenticule as well as the depth of implantation and corneal wound healing response probably determines the final corneal refractive power. Our principal purpose in current study was to increase and stabilize corneal thickness, and to prepare it for correction of total refractive error including lower and higher order aberrations by customized or T-cat PRK. In three patients we had to perform DALK after three to six months due to patient dissatisfaction. The main cause of visual deterioration seems to be graft folds and wrinkling in the visual axis. Another reason could be a thick layer of donor tissue in the central 3 mm zone. Two patients agreed to wait longer for a second procedure. Corneal thickness and power were stabilized after one year and remained stable until 55 and 51 months after implantation. The lenticule folds and wrinkling were significantly decreased on the last visit and their clarity was the same as the recipient stromal cornea.

In summary, the current report demonstrated the feasibility of Femtosecnd Laser-assisted Allogenic Stromal Keratoplasty Without and With Excimer Laser-assisted Donor Keratomileusis, briefly “FASK” and “FASK plus EDK” in the management of KCN; with superior refractive and keratometric outcomes in the “FASK Plus EDK” group. Nevertheless, visual outcomes were not satisfactory in three of five patients several months after surgery which indicates the long time for visual recovery and stabilization by both of these methods. It means that patients need to wait more than one or two years for visual recovery which is not acceptable for most of them. Considering limitations of the current series including small sample size due to strict ethical issues, this new surgical modality could open a new path to treatment of primary corneal ectasia, reducing the need for conventional keratoplasties. Although using a thick center lenticule (what we used) did not work well, our results provide encouraging preliminary information for the design of future studies on allogenic intrastromal rings, paracentral segments or lenticules with a thinner center.

##  Financial Support and Sponsorship

Nil.

##  Conflicts of Interest

There are no conflicts of interest.
